# The effects of stem length and core placement on shRNA activity

**DOI:** 10.1186/1471-2199-12-34

**Published:** 2011-08-08

**Authors:** Glen J Mcintyre, Yi-Hsin Yu, Mehnaaz Lomas, Gregory C Fanning

**Affiliations:** 1 Johnson and Johnson Research Pty Ltd, Level 4 Biomedical Building, 1 Central Avenue, Australian Technology Park, Eveleigh, NSW, 1430, Australia; 2 School of Biotechnology and Biomolecular Sciences, The University of New South Wales, Sydney, NSW 2052, Australia; 3 Tibotec BVBA, Gen De Wittelaan L 11 B3, 2800 Mechelen, Belgium

## Abstract

**Background:**

Expressed short hairpin RNAs (shRNA) used in mammalian RNA interference (RNAi) are often designed around a specific short interfering RNA (siRNA) core. Whilst there are algorithms to aid siRNA design, hairpin-specific characteristics such as stem-length and siRNA core placement within the stem are not well defined.

**Results:**

Using more than 91 hairpins designed against HIV-1 Tat and Vpu, we investigated the influence of both of these factors on suppressive activity, and found that stem length does not correspond with predictable changes in suppressive activity. We also detected multiple processed products for all stem lengths tested. However, the entire length of the hairpin stem was not equally processed into active products. As such, the placement of the siRNA core at the base terminus was critical for activity.

**Conclusion:**

We conclude that there is no fixed correlation between stem length and suppressive activity. Instead, core selection and placement likely have a greater influence on the effectiveness of shRNA-based silencing.

## Background

RNA interference (RNAi) in mammalian cells is a post-transcriptional gene silencing mechanism that functions to regulate gene expression via small hairpin-like dsRNA molecules called MicroRNA (miRNA). miRNA precursors (pri-miRNA) are first processed in the nucleus by a Drosha complex cleaving ~ 22 bp back from the stem-loop junction (the loop terminus) to release a 60 - 80 nucleotide (nt.) hairpin (pre-miRNA) [1]. In the cytoplasm, Dicer next cleaves from the opposite end (the base terminus), removing the loop to release a small RNA duplex of ~ 21 bp (the mature miRNA) [2,3]. The duplex is then unwound and loaded into the RNA induced silencing complex (RISC) in a process that favors one of the two strands (the guide strand) based on a difference in thermodynamic stability at the ends of the duplex [4]. The guide strand directs the RISC to bind target RNA, and in the context of mammalian RNAi, generally results in target degradation if the match is perfect, or translational repression if the match is imperfect.

**Figure 1:**
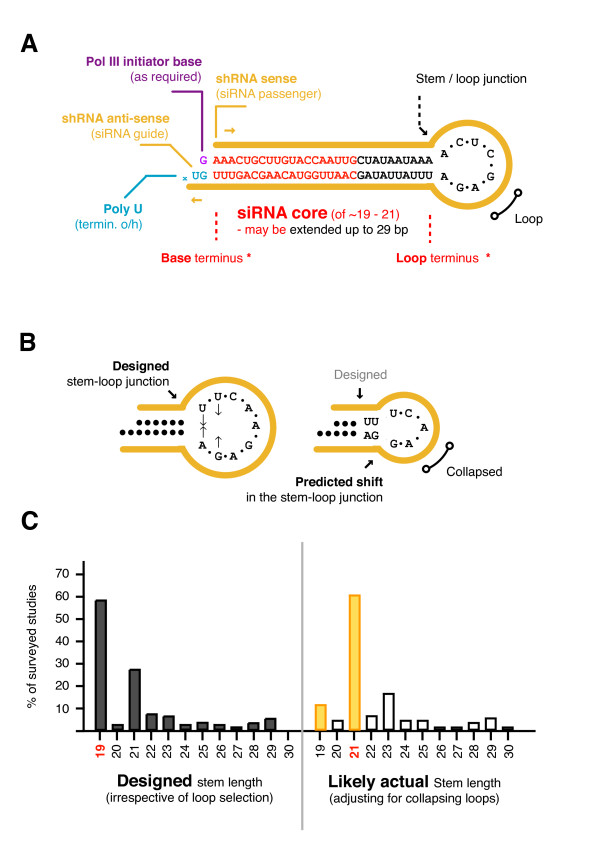
**Hairpin anatomy and the most common stem lengths**. (**A**) Typical hairpin design begins with an optimal siRNA core that is extended in one or both directions to a total stem length of 19 to 29 bp. The 3' end of the upper siRNA strand is connected to the 5' end of the lower siRNA strand by a loop sequence. The shRNA stem length is defined as the stretch of sequence between the terminally paired nucleotides. The upper strand of the stem is the 'sense' strand which is designed to give rise to the siRNA 'passenger' strand. The lower strand is the 'anti-sense' strand and is designed to give rise to the siRNA 'guide' strand. (*) The stem region towards the free end of the hairpin is referred to as the base terminus whereas the stem region towards to the loop end is referred to as the loop terminus. The point at which the stem meets the loop is referred to as the stem-loop junction. (**B**) A commonly used loop sequence (UUCAAGAGA) (used in 60% of surveyed studies) is predicted to internally pair (UU.. to ..GA) resulting in an unintended shift in the stem-loop junction. (**C**) 101 studies employing expressed shRNA were surveyed and each hairpin was scored for stem length. The stem lengths were found to range from 19 - 29 bp, with the most commonly designed stem length being 19 bp (58% of all hairpins). When designed stem lengths are adjusted for additional loop sequence the most common length is 21 bp (60%).

RNAi can also be co-opted by delivering synthetic short interfering RNA (siRNA) duplexes of ~ 19 - 21 bp that are loaded directly into RISC [5,6]. Alternatiely, short hairpin RNA (shRNA) (typically < 30 bp) can be expressed from polymerase III promoters [7-14] to be subsequently processed. shRNA design often occurs by the addition of a loop to an optimally designed siRNA core, which may also be extended to increase the stem length (Figure 1A). Whilst there are now many siRNA design guidelines [4,15], the additional parameters specific to shRNAs - such as stem length, core position, flanking and loop sequence, are not so well-defined.

While stem typically varies between 19 and 29 bp, few studies have investigated its importance for suppressive activity and these were neither overlapping in scope nor corroborative in conclusion [16-19]. Some of the conclusions include: poor short hairpins (19 bp) can be improved with an increase in stem length (28 bp) [16], longer hairpins (25 - 29 bp) are simply more active [17], and shorter hairpins (21 bp) are better [18]. The situation is clearly unclear. Additionally, there are more recent claims that longer synthetic siRNA/shRNA duplexes (27 - 29 bp) are better due to 'improved Dicer processing' [20-22]. But, this may not apply to expressed shRNA as the synthetic approach may not incorporate processing events upstream of Dicer recognition. Despite this the high-profile nature of this research has lead to a general expectation that increasing the stem length of an expressed shRNA will also lead to enhanced processing and therefore enhanced suppression [23,24]. Our work and other recent reports now challenge this expectation [19,25].

In cases where the selected stem length exceeds the length of the designed siRNA core, then the placement of the core within the shRNA must also be considered. In this sense the terms 'core' and 'placement' refer to a predetermined siRNA sequence from which shRNAs are often derived, and its position within the (often) longer shRNA stem. Current understanding from *in vitro *Dicer studies is that RNA duplex processing occurs from the termini [26-28], but again, whether this is equally applicable for expressed shRNA is currently unknown. Although placement of the core has varied for traditional shRNAs, including positions 1 [29], 2 [22], 3 [30], 6 [16] and others (as measured from the base terminus), there has been no systematic study investigating its importance for subsequent activity.

In this study we asked how is the suppressive activity of expressed shRNA altered when changing the length of the stem, and how does suppressive activity relate to the placement of a predetermined siRNA core within the shRNA stem? To answer these questions we tested more than 91 hairpins targeting HIV-1 Tat or Vpu, varying in both stem length and sequence composition. We found no fixed correlation between stem length and suppressive activity, and showed that core placement at the base terminus is critical for activity.

## Results

### The most common stem lengths are 19 and 21 bp

We first surveyed 101 expressed shRNA studies to determine the most commonly used hairpin stem lengths and loop sequences (Additional file 1). All stem lengths ranged from 19 to 29 base-pairs (bp), with 19 bp (used in 58% of studies) and 21 bp (27%) the most common. It was also found that ~ 60% of studies use the same 9 base loop (UUCAAGAGA) first reported in one of the earliest shRNA studies [7].

Closer analysis of this loop reveals that it is predicted to pair internally (UU.. to ..GA) resulting in a collapsed loop size of 5 bases (CAAGA) and a stem which is extended by 2 bp (Figure 1B) [31-33]. Therefore, when adjusting the surveyed stem lengths for this extra sequence, the frequency of 19 bp stems drops to 11% and the frequency of 21 bp stems rises to 60%, making 21 bp the most common length (Figure 1C).

### Short and long shRNAs are both potent suppressors

To investigate the effects of increasing stem length on shRNA activity, we designed a set of 17 hairpins with 15, 17 - 29, 33, 37, and 41 bp stem lengths (Table 1 and Additional file 1). This included every length between the common bounds of 19 and 29 bp, plus some additional shorter and longer ones. All were designed to target Tat (from HIV-1_NL4-3_; Genbank: AF324493), initiating from a common 5' position (nt. #56 of the target gene), with the stem/target sequence extending in the 3' direction. All other factors of the hairpin design were kept constant. Suppressive activities were measured as a reduction in GFP fluorescence from a target-fusion reporter, after transient expression of both the hairpin and reporter(s) in HEK293a cells, relative to an empty expression vector control (expressing no hairpin). An additional non-targeted reporter was included so as to measure and normalize for non-specific effects. Non-specific activity is represented by the normalization factor in fold-changes relative to the empty expression vector control, where a factor of 1 (shown as red bars) represents no non-specific activity.

**Table 1:** shRNA sense-stem/target sequences

cnt	Hairpin	Target sequence covered (sense) ^a, b, c^
**16**	Tat56 (15 - 41) ^d^	**A**AACUGCUUGUACC**A^15^**AU**U^18^G^19^C^20^U^21^A^22^****U^23^U^24^G^25^U^26^A^27^A^28^A^29^**gAG **U^33^**GUU**G^37^**CUU**U^41^**
**6**	Vpu10 (19 - 29)	**A**UAAUAGUAGCAAUAGUA**G^19^**C**A^21^**U**U^23^**A**G^25^**U**A^27^**G**U^29^**
**6**	Vpu51 (19 - 29)	**A**GCAAUAGUUGUGUGGUC**C^19^**A**U^21^**A**G^23^**U**A^25^**A**U^27^**C**A^29^**
**6**	Vpu127 (19 - 29)	**G**AUAGACUAAUAGAAAGA**G^19^**C**A^21^**G**A^23^**A**G^25^**A**C^27^**A**G^29^**
**8**	Vpu158 (17 - 29)	**G**CAAUGAGAGUGAAGG**A^17^G^18^A^19^**A**G^21^**U**A^23^**U**C^25^**A**G^27^**C**A^29^**
**2**	Tat3 (21, 29)	**G**GAGCCAGUAGAUCCUAGAC**U^21^**AGAGCCC**U^29^**
**4**	Tat22/24 (21. 29)	**C**U**A**GAGCCCUGGAAGCAUCC**U^21^**G**G^21^**AAGUC**A^29^**G**C^29^**
**2**	Tat94 (21, 29)	**U**UUCAUUGCCAAGUUUGUUU**C^21^**AUAACAA**A^29^**
**2**	Tat144 (21, 29)	**G**CAGGAAGAAGCGGAGACAG**C^21^**GACGAAG**A^29^**
**2**	Tat165 (21, 29)	**G**ACGAAGAGCUCAUCAGAAC**A^21^**GUCAGAC**U^29^**
**4**	Tat181/187 (21, 29)	**A**ACAGU**C**AGACUCAUCAAGC**U^21^**UCUCU**A^21^**UC**A^29^**AAGC**A^29^**
**5**	(Tat56) 19+10 to 27+2	AAACUGCUUGUACCAAUUG**^19^**- {**gauaacauuu^+10 ^**| CU**^21^uaacauuu^+8 ^**| CUAU**^23^acauuu^+6 ^**| CUAUUG**^25^auuu^+4 ^**| CUAUUGUA**^27^uu^+2^**}
**5**	(Tat56) 10+19 to 2+27	{**uuugacgaac^10+ ^**| **uuugacga^8+^**UG | **uuugac^6+^**CUUG | **uuug^4+^**UGCUUG | **uu^2+^**ACUGCUUG} -**U^11^**ACCAAUUGCUAUUGUAA**A^29^**
**1**	Tat59 21	**C**UGCUUGUACCAAUUGCUAU**U^21^**
**9**	(Tat59) 0-21-8 to 8-21-0	{_**^0 ^**| **u^1 ^**| **uu^2 ^**| **uuu^3 ^**| **guuu^4 ^**| **aguuu^5 ^**| **gaguuu^6 ^**| **ggaguuu^7 ^**| **cggaguuu^8^**} -CUGCUUGUACCAAUUGCUAU**U^(Tat59-21)^**- {**cauuuggc^8 ^**| **cauuugg^7 ^**| **cauuug^6 ^**| **cauuu^5 ^**| **cauu^4 ^**| **cau^3 ^**| **ca^2 ^**| **c^1 ^**| **_^0^**}
**6**	(Tat56) 23+6 {v2 - v7}	**A**AACUGCUUGUACCAAUUGCUAU**^23^**- { **gggggg^v2 ^**| **cccccc^v3 ^**| **auauau^v4 ^**| **gcgcgc^v5 ^**| **gacugu^v6 ^**| **acaguc^v7 ^**}
**8**	(Tat56) 23+1 to 23+18 ^e^	**A**AACUGCUUGUACCAAUUGCUAU**^23^**- {**a^24^c^25^a^26^t^27^t^28^tgtca^33^aacg^37^aaag^41^**}
**92**		

**cnt **: (count) the total number of new hairpins in each set (excluding hairpins already counted as a part of earlier sets).

**^a ^**: terminal nucleotides for each shRNA target/sense stem are indicated in **bold**, with the stem length indicated for each shRNA made.

**^b ^**: Sense stem sequence mismatched to the target is shown in **lowercase**. { *a | b | c | etc*. }: Continuing sequence where only one of the options was incorporated per shRNA.

**^c ^**: The binding region for the Tat56 19 nt. probe (for Northerns) is underlined.

**^d ^**: shRNA stem position 30 was changed in Tat56 33, 37 and 41 from 'A' to 'G' to prevent premature transcirptional termination from a run of 4 'T's.

**Figure 2:**
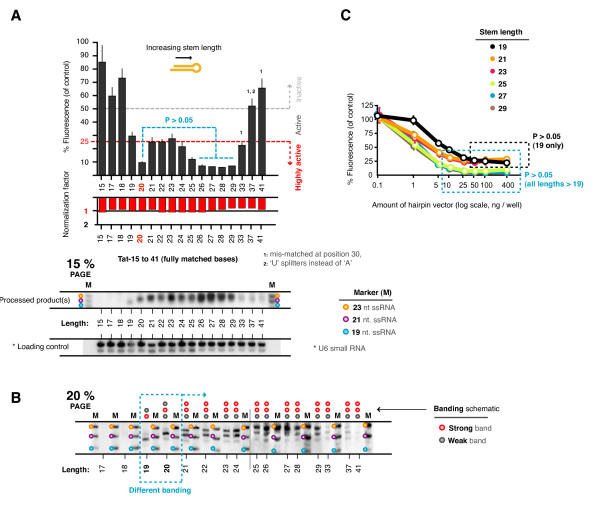
**Short and long shRNAs are both potent suppressors**. (**A**) The suppressive activities of a set of 17 related hairpins with 15, 17 - 29, 33, 37, and 41 bp stem lengths were tested. n.b. for hairpins longer than 29 bp, position 30 (1) was altered to a 'G' to prevent a run of 'T's which would have resulted in premature termination of transcription, however, this was not expected to impact on suppressive activity. Three sets of data are shown; specific suppressive activity in the top (as a decrease in fluorescence of the GFP-target fusion), non-specific shRNA effects in the second row graph (represented by the normalization factor), and standard expression anlysis of the processed siRNA products (15% PAGE) on the third row. (**B**) Additional high resolution northern analysis (20% PAGE) for the same hairpins. (**C**) 293a cells were transfected with increasing amounts of hairpin vector from 0.1 ng to 400 ng, for the Tat56-19, 21, 23, 25, 27 and 29 bp hairpins. Each reaction was supplemented with the appropriate amount of empty expression vector to keep the total expression vector DNA delivered at 400 ng. All activity values are averages from at least 3 independently repeated experiments with 95% Confidence Intervals (CI) shown.

Hairpins shorter than 19 bp and longer than 33 bp showed no notable activity (Figure 2A). Those from 19 - 33 bp were all active, with a progressive increase in activity in lengths from 21 - 23 to 26 - 29 bp. However, the potency of the shorter hairpin of 20 bp was a notable exception to this progression, with high suppressive activity indistinguishable from the 26 - 29 bp hairpins (P > 0.05). Expression analysis (on 15% PAGE gels) confirmed the presence of the expected products for hairpins across the range of 19 - 29 bp stem lengths. Products for hairpins shorter than 19 bp were not detected. Hairpins longer than 29 bp had low levels of detectable product which may have been due to inefficient processing, or less of the probe-specific sequence being incorporated into the active product. We also noticed that the processed product(s) for the 19 bp hairpin appeared smaller than those of the other hairpins and thus we conducted additional high-resolution analysis (20% PAGE) (Figure 2B). Unexpectedly, this showed that many hairpins were processed into multiple products, and clearly showed that the products of the 19 and 20 bp hairpins were smaller than those from the longer hairpins. Hairpins shorter than 21 bp had a single predominant product, whereas those of 21 bp and longer had 2. Importantly, these experiments showed that shorter hairpins can be just as potent as their longer counterparts.

### Hairpins of different lengths are similarly dose-dependent

To investigate whether the activity differences for hairpins of different length had different dose-dependences, we looked at a sub-set of these hairpins (19, 21, 23, 25, 27 and 29 bp) at various dosages. Suppressive activities were measured using hairpin expression vector amounts from 0.1 - 400 ng (Figure 2C). Each sample was supplemented with the appropriate amount of empty expression vector to keep the total DNA delivered constant, thus maintaining consistent transfection conditions between samples. The dose-effect relationships were similar for all hairpin lengths except for the 19 bp hairpin for which higher doses were required for half-maximal and maximal activity. For all lengths tested there was a dosage at which the system was saturated and no further increase in suppressive activity was achievable by adding more vector alone.

### There is no fixed correlation between stem length and activity

**Figure 3:**
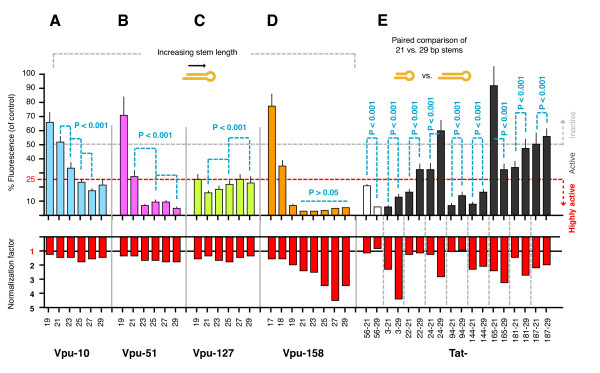
**There is no fixed correlation between stem length and activity**. (**A - D**) Four extra hairpin sets of 19, 21, 23, 25, 27 and 29 bp stems were designed to target Vpu, initiating from nucleotide positions 10, 51, 127 and 158 (relative to the start of the Vpu gene). The Vpu 158 set was expanded to include 17 and 18 bp stems. (**E**) Additional Tat hairpin pairs were designed to compare the activity of the most commonly employed short and long stem lengths (21 cf. 29 bp), and included the Tat56 21 and 29 bp hairpins. All values are averages from at least 3 independently repeated experiments with 95% Confidence Intervals (CI) shown.

We further tested the relationship between stem length and suppressive activity by looking at different targets, including another 3 target sets of 19, 21, 23, 25, 27, and 29 bp hairpins directed to different regions of another HIV-1_NL4-3 _gene, Vpu (Figure 3A-D). As before, all hairpins within each set initiated from a common 5' terminus. For 2 sets we observed a trend of increasing activity with stem length (Vpu 10 and 51, P < 0.001), in another we saw the opposite trend (Vpu 127, P < 0.001), and in the final set all hairpins were highly active (Vpu 158, P > 0.05). We thus expanded this last set to include 17 and 18 bp versions; lengths generally considered too short to be effective substrates for Dicer processing [6,22,34], and found that activity could be retained in a stem length of 18 bp. We also tested another 8 matched Tat hairpin pairs that were available to us. Each of these pairs comprised the most common short (21 bp) and long (29 bp) hairpins, with each pair targeting a different region of Tat (Figure 3E). The short hairpins ranged from highly active to inactive, and 7 of the 8 pairs showed a significant loss in suppressive activity with an increase in stem length, irrespective of the activity level of the short hairpin (P < 0.001). Thus, overall we conclude that there is no fixed correlation between stem length and activity.

### Target-matched sequence at the base terminus is critical for activity

**Figure 4:**
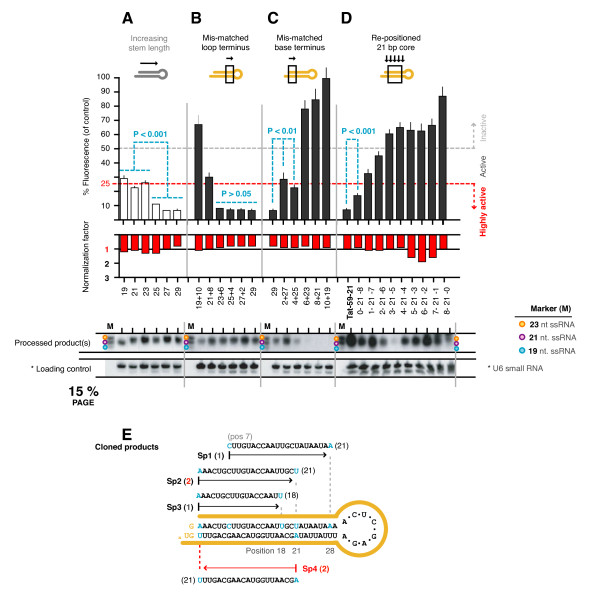
**Target-matched sequence at the base terminus is critical for activity**. All hairpins were designed around position 56 of Tat (or 59 as indicated in the final set). (**A**) The same Tat56-19, 21, 23, 25, 27 and 29 bp hairpins as tested before, representing the span of commonly employed stem lengths, were reused here for comparison (shown with open bars). (**B - C**) For a constant stem length of 29 bp, inverted sequence was introduced at either the loop terminus or base terminus in 2 bp increments. (**D**) A 21 bp siRNA core was progressively re-positioned at each of 9 possible positions in the stem of a 29 bp hairpin. All values are averages from at least 3 independently repeated experiments with 95% Confidence Intervals (CI) shown. (**E**) The Tat56-29 shRNA was transiently expressed in 293a cells from which small RNA species were extracted and cloned. One hundred and thirty four individual ssRNA species were cloned and six were found that originated from the Tat56-29 hairpin. The remaining sequences were bacterial in origin or unidentified.

Given that the most common stem lengths are bound between 19 and 29 bp (Figure 4A), we created several sets of 29 bp hairpins that differed in the placement and amount of sequence that was homologous to the target in odd-length increments from 19 to 29 bp to study core positioning. These were all created around the same target as before (Tat56). In the first set, inverted sequence was introduced at the loop terminus in 2 bp increments so as to be mismatched to the target, but to retain an identical thermodynamic profile ('A' to 'T', 'G' to 'C' and vice versa) (Figure 4B). Inclusion of target-mismatched bases at the loop terminus was well tolerated with no significant loss in suppressive activity for hairpins with 23 bp or more of matched sequence to the target (P > 0.05). Hairpins containing less than 23 target-matched bases had impaired suppressive activity, and the hairpin with only 19 bp of target-matched sequence was non-functional. Processed products were detected as expected.

Inverted sequence was similarly introduced in the second set, but from the opposite terminus (Figure 4C). Unlike the first set, even the smallest inclusion of target-mismatched sequence impaired activity (P < 0.01). The slight increase in activity with 4 mismatches, compared to 2 mismatches (P < 0.01), occurred consistently (across several separate experiments), but for unknown reasons. Hairpins with 6 or more target-mismatched base pairs at the base terminus were non-functional. Processed products were not detected for hairpins with only 21 and 19 bp of target-matched sequence at the loop terminus; however, this was most likely due to inadequate homology with the probe. Overall, the results suggested that for hairpins with 29 bp stems, the primary (or sole) agent was derived from positions 1 - 2 to 22 - 23 of the paired stem, and was therefore 21, 22, or 23 bases long, which was within the size range of the processed products perviously detected.

### The entire length of a 29 bp hairpin stem is not equally processed

To test the importance of core placement in a second way, another set of 29 bp hairpins was made with an active 21 bp siRNA core placed at each of the 9 possible positions (Figure 4D). This experiment thus tests the outcome of placing the core at all of the different positions possible within a given shRNA stem length. To avoid confusion in following this work, remember that the 'core' corresponds to a predetermined siRNA, and in this case, one already verified as highly active. As before, the sequence outside the core was inverted to be mismatched to the target. Progressive repositioning of the core towards the loop terminus correlated with reduced activity. The most active 29 bp hairpin had the core positioned at the base terminus (p1) confirming for longer hairpins that it is the sequence at the base terminus that is the primary contributor to suppressive activity. However, we also found that the 21 bp control hairpin (composed of just the 21 bp core sequence) was more active than the equivalent 29 bp hairpin (P < 0.001) (composed of the 21 bp core plus 8 bp of inverted sequence; p1). The processed products were detected at approximately equivalent levels between all variants with the exception of the 3-21-5 variant. The reasons for this exception were unclear, but given that it did not correlate to a relative change in the measured suppressive activity we considered that it is was most likely an artifact of the detection process, e.g. inefficient probe binding. The activity results support the conclusion that the entire length of the stem is not equally processed into multiple siRNA species. These findings further support the idea that it is the base terminus from which processing occurs, such that as we progressively moved our core along (from the base to the loop terminus) we were most likely creating processed (siRNA) products with decreasing homology to the target (at the 5' end of the upper strand of the processed duplex).

### Most cloned processed products originate from the base terminus

The processed products for the fully-matched 29 bp hairpin (Tat56-29) were cloned (in-house) to verify the notion that the base terminus was giving rise to the most prominent processed product. Cells were transfected with the hairpin expression vector and total RNA was isolated 48 hours later. Short RNA species were selectively isolated (using PAGE separation) and cloned using the Lau and Bartel small RNA cloning protocol [35]. Six species that aligned to the hairpin were identified from 134 total species cloned and sequenced (Figure 4E). Five of these, Sp2 (x2), 3 and 4 (x2) originated from the base terminus of hairpin between positions 1 and 21, and one, Sp1 came from positions 7 - 28. Though the number of relevant sequences recovered were few, these confirmed that multiple different-length products can be generated from a single shRNA, with a bias towards processing from the base terminus (for a 29 bp hairpin).

### A 23 bp hairpin can be improved by an increase in stem length

**Figure 5:**
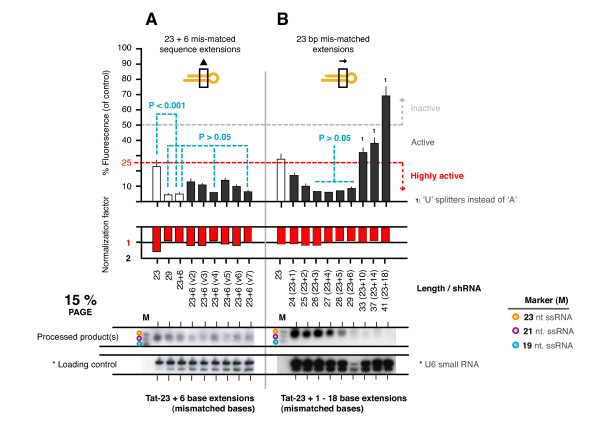
**A 23 bp hairpin can be improved by an increase in stem length**. (**A**) The last 6 bp of a 29 bp hairpin were replaced with extensions of varying GC contents. (**B**) An additional set of hairpins of 23 - 29, 33, 37 and 41 bp stems in which only the first 23 bp was matched to the target, with the remaining sequence inverted were created and tested. All values are averages from at least 3 independently repeated experiments with 95% Confidence Intervals (CI) shown.

On comparing all data sets, we noted that the activity of the 29 bp hairpin composed of 23 bp of target-matched sequence at the base terminus, with 6 bp of inverted sequence at the loop terminus, was significantly more active than the corresponding hairpin with a perfectly target-matched 23 bp stem (compare 23 to 23 + 6). This suggests that the additional 6 bp increased the activity of the hairpin purely as a function of increased stem length and not target-specificity. Therefore, another set of 29 bp hairpins was made with 23 bp of target-matched sequence at the base terminus plus 6 bp sequence extensions with alternative sequence compositions (Figure 5A). All variants remained highly active, irrespective of the sequence composition. However, the activities of hairpins with weakly bound extensions (i.e. more A:T pairs) were not significantly different from the original hairpin (P > 0.05). By contrast, the activities of the hairpins with more strongly bound extensions (i.e. more G:C pairs) were significantly reduced (P < 0.001). We extended this experiment by creating an additional set of hairpins of 23 - 29, 33, 37 and 41 bp stems in which only the first 23 bp was matched to the target, with the remaining sequence inverted (Figure 5B). The suppressive activity of these partially matched hairpins closely followed that of their fully matched counterparts tested earlier (P > 0.05 for lengths 25 - 29 bp across the two sets), further suggesting that increasing stem length beyond 23 bp, irrespective of target specificity, can enhance activity. This however, is not necessarily always true of other targets, nor of comparisons between shorter hairpins (< 23 bp) and 29 bp hairpins, as evident from our prior hairpin sets.

## Discussion

We analyzed more than 91 expressed shRNAs that varied in target site, stem length, and sequence composition to study the effects of changing stem length and core placement on suppressive activity. In contrast to *in vitro *studies [23,24], which focus on isolated points of the RNAi processing pathway, our hairpins were subject to every cellular processing step, increasing the relevance of our findings to present shRNA use. Our results conclusively show that there is no fixed correlation between hairpin stem length and suppressive activity. In some cases activity was increased by the addition of extra sequence (which need not be target-matched), yet in others it was not. We found that the placement of the designed siRNA core at the base terminus was critical for activity, as the entire length of the hairpin stem was not equally processed into active products. We found highly active hairpins from all stem lengths tested between 19 to 29 bp, plus an active 18 bp hairpin as well. This is a very interesting finding, as in conjunction with the recent report that synthetic siRNA triggers of only 16 bp are still effective silencers [36], it suggests that the minimal effective duplex size may be smaller than previously thought.

Taken together, our results lead us to speculate that shRNAs may be processed differently depending on stem length, with divisions at ~ 20 bp and ~ 29 bp. Those shorter than 21 bp may bypass one or more processing steps, which is supported by reports that both 19 bp siRNA duplexes [6] and 19 bp synthetic shRNA [22] are not processed by Dicer, yet they retain *in vivo *activity. This, however, may also relate to loop size (in the context of shRNAs), where shorter loops of ~ 4 nt. (cf. 8 - 9 nt.) may be less likely to engage Dicer [22,25]. Our hairpins of 21 to ~ 29 bp seem to have a common position of processing relative to the base terminus which is consistent with the known mechanism of Dicer [26,28]. *In vitro *studies have shown that the exact point of Dicer cleavage can vary, yielding more than one product differing by 1 - 2 bp [20,27]. This provides a mechanism for the multiple products we observed here. In contrast to Dicer, Drosha processes relative to the opposite end; measuring back from the stem-loop junction [1,37]. Although Drosha (or a Drosha complex) requires a large loop for efficient processing (length ≥ 10 bases), in its absence it may separate an adjacent portion of the stem to attain it [1]. This supports our interpretation that the stem of 29 bp hairpins may be unwound outside the active ~ 23 bp base-terminus region. We stress, however, that these are only speculative interpretations to position our findings in the context of the current understanding of the field. They, of course, require testing using a number of knockout-type studies (e.g. Dicer/Drosha ) and extensive deep-sequencing of different length shRNAs (e.g. Illumina/454 sequencing methods). It would also be very revealing to do detailed follow-up work on further data sets, such as with the 4 Vpu data sets, and include Northern and sequencing analysis to begin to assemble some guidelines for future shRNA constructions.

Presumed processing by either Dicer or both Drosha and Dicer is now generally considered to result in both greater siRNA production and potency [16,22,38,39]. Although this may allow for activity at lower DNA concentrations, which may be beneficial for reducing non-specific effects [40,41], we surmise that it does not reliably dictate increased shRNA activity. Instead, our data leads us to conclude that the primary determinant of activity is inherent to the sequence of the processed product(s) - regardless of the mode of processing. Furthermore, there are claims that coordinated processing by both Drosha and Dicer will reduce the heterogeneity of the processed products [39,42,43]. Our data shows that for longer lengths, with presumably increased processing (applicable for stem lengths ≥ 21 bp), there are more products formed and thus increased processing alone is not a predictor for a more defined product. Given that it is the product identity that determines activity, multiple products could cause competition such that the activity of a potent suppressor may be 'diluted', a view shared by others [27]. Some suggest that it may be possible to engage both Drosha and Dicer to yield a single defined product by using 'second-generation' hairpin designs that more closely replicate specific microRNA structures (e.g. miR30) [38,44-47]. However, more recent data suggests that this may not always be the case [48]. Indeed, there is now mounting evidence which challenges the idea that second generation designs are improvements on standard shRNAs [19,25,49].

Rather than longer hairpins (of 29 bp), our results show that shorter hairpins (stem length ≤ 20 bp) may be better for generating single products. This could have the added advantage of reducing competition (for Drosha and Dicer) with natural small RNAs involved in cell regulation - a commonly voiced concern when 'hijacking' the RNAi pathway [42,50-52]. Moreover, it is possible that of all stem lengths, those shorter than 21 bp are the least likely to induce non-specific effects [41]. However, as we noted, 81% of hairpins currently designed with 19 bp stems actually have 21 bp stems due to a collapsing loop. If our findings hold true, then ~ 85% of studies are using hairpins that yield more products than their shorter counterparts. These products may differ by only 1 or 2 bases but we, and others, have shown that minor sequence changes even as small as 1 - 2 bases can have large effects on suppressive activity [15,53,54]. Furthermore, it is possible that the products of 19 and 20 bp stems incorporate additional sequence derived from the loop or flanking regions [32]. Optimal use of short hairpins requires extra consideration of the structure and composition of the loop, and possibly flanking sequence. The presence of multiple products, the potential incorporation of extra-stem sequence and the unequal processing of the entire stem length may, in-part, explain why it is often difficult to retain siRNA activity when constructing the corresponding shRNA.

In addition to stem length and core placement, there are also several other shRNA-specific variables that have recently been reported on. The work of Li *et. al*., asking some similar questions to those here and extensively comparing short and long shRNAs, supports our findings by also showing that shorter hairpins can be highly effective silencers [25]. They also looked at loop sequence and the influence that loop size has on suppressive activity. They concluded that short loops of 4 nt. may curtail the activity of otherwise effective core sequences. Longer loops of ~ 9 nt. were shown to be generally more effective - though, most importantly, it should be noted that their 'longer' 9 nt. loop was the collapsing type. This is most interesting, as in-effect their 'shorter' shRNAs may have been ~ 21 bp stems with only a 5 nt. loop - not 'truly' short hairpins nor 'longer' loops by our definitions. Others also report that the suppressive activities of shorter hairpins (of ~ 19 bp) may be more susceptible to the negative effects of shorter loops than those of longer stem lengths (albeit for synthetic shRNAs) [55]. These findings are interesting and warrant further investigation, especially given that our survey indicates that the majority of studies may be using short loop sizes of ~ 5 nt. Finally, asymmetric strand biasing is yet another design parameter which has recently been shown to influence resulting suppressive activity, and therefore one which should also be carefully considered in shRNA constructions [49,56].

## Conclusion

In summary, we found that although the processing of a hairpin is dependent on its stem length, the activity of a hairpin is primarily dependent on the sequence of its processed product(s). The comparison of hairpins shorter or longer than 21 bp loses meaning when considering that it is most likely a comparison of different siRNAs. We conclude that there is no fixed correlation between stem length and suppressive activity, though in some cases the activity of hairpins of at least 23 bp may be improved by stem extensions. From a purely activity point-of-view, neither short nor long hairpins should be discounted as potentially potent suppressors. There may, however, be some other advantages to using shorter hairpins (< 21 bp) over longer ones (e.g. fewer products produced). Instead of stem length though, it is siRNA core design and placement that are most likely to have the greatest influence on ensuing suppressive activity.

## Methods

### shRNA vector construction

All hairpins were expressed from a derivative of pSilencer 3.0-H1 (Ambion) via a human H1 polymerase III (pol III) promoter. Each shRNA insert was constructed using either annealed complementary oligonucleotides (oligos) or Phi-29 based primer extension [33]. All shRNA vectors were propagated in GT116 E. coli cells (a cell line specifically developed for the replication of hairpin containing vectors) (Invivogen). DNA was extracted (Hi-speed Maxi-prep Kit, Qiagen) and quantitated in triplicate. All shRNA expression constructs were restriction enzyme digested using a site engineered into the loop before sequence confirmation to enable automated sequencing of hairpin vectors possessing reaction-inhibiting secondary structure [33].

### shRNA design

We developed a standard design (so that comparable hairpins differed only in the paired stem region) which included a 'G' at the +1 position (to equalize initiation of pol III transcription), a standard loop (ACUCGAGA, based on a *Xho *I site to allow vector sequencing, but designed to remain in an 'open' configuration), and a final 'G' (to prevent premature termination by an early run of 'T's) prior to the H1 promoter termination signal (TTTTTTGGA). It was expected that pol III termination would add a variable number of 'U's to the 3' end of each hairpin, but the exact number of which was unknown as there are conflicting reports of anywhere from 1 - 6 residues being added (2 U's [7,8,17], ≤ 4 U's [12], 4 U's [10,14], ≤ 5 U's [13,27], 4 - 6 U's [57]). Hairpins were designed to target HIV-1 Tat or Vpu based on previously described siRNA sequences [58] or siRNA design guidelines [4,15] (Additional file 1).

### Assay vector construction

The assay vectors, pd4EGFP-sTat (target vector), pd4EGFP-sVpu (target vector) and pAsRed1-sVif (control vector) were constructed using EGFP (from pd4-d4EGFP-N1, BD Biosciences), AsRed1 (from pAsRed1-C1, BD Biosciences) and HIV-1 sequences from variant NL4-3 [Genbank:AF324493]. The complete target sequences can be found in Additional file 1. Each vector was designed to produce a single mRNA transcript comprising the fluorescent protein fused to a downstream HIV-1 gene sequence but separated by multiple stop codons to ensure that only the first domain would be translated (the fluorescent protein).

### shRNA activity assay

HEK293a cells (sourced from the American Type Culture Collection) were seeded at a density of 5 × 10^5 ^cells per well (6 well plates; 2 ml of medium). Cells were transfected 1 day later using 1 μg of total DNA (400 ng of shRNA expression vector, 300 ng of target vector and 300 ng of control vector) with 4 μl of Lipofectamine 2000 (Invitrogen) in OptiMEM (Invitrogen) to a total volume of 100 μl/well. Cells were analyzed by flow cytometry 2 days later (FACsCalibur, BD Bioscience). Target-specific suppression was measured as a decrease in green fluorescence (FL1 channel) and non-specific effects were measured as a change in red fluorescence (FL2 channel). The Fluorescence Index (FI) of cells in each channel was calculated by multiplying the geo mean of fluorescence by the percentage of cells that were fluorescent (only those cells gated above background). The FI of FL1 (green, target-specific activity) was normalized to remove non-specific effects (FI of FL1 normalized to the FI of FL2) and was expressed as a percentage of the FI of cells transfected only with the control vector that expressed no hairpin. The normalization factor was shown as a relative measure of non-specific shRNA activity (a value of 1 equals no non-specific activity, i.e. the measured non-specific activity was identical to that measured for the control vector that expressed no hairpin). Each sample was analyzed in triplicate and each experiment was repeated at least 3 times with 95% confidence intervals shown. Every experiment included a mock transfection (i.e. no DNA) and an off-target hairpin control (to verify that on-target hairpin suppression was sequence-specific), both of which behaved as expected and both of which were omitted from the graphs for clarity.

### Northern blot analysis

Following cytometric analysis, RNA was extracted from each sample using Trizol (Invitrogen). Total RNA (5 - 10 μg in 10 μl) was separated under denaturing conditions using 15 - 20% TBE urea polyacrylamide gel electrophoresis (PAGE), was transferred to nylon membranes at 30 V limiting for 60 - 90 min and cross-linked to the membrane. Membranes were hybridized overnight at 42°C using OligoHyb (Ambion) and 200 ng of a 19 base, 3' biotin end-labeled DNA probe (Sigma-Genosys) designed to bind both the shRNA anti-sense strand (hairpin precursor) and the siRNA guide strand (processed product). Probe binding was detected by streptavidin/alkaline phospatase conjugation using either the Brightstar Biodetect kit (Ambion), or the Phototope detection kit (New England Biolabs). Membranes were stripped by standard procedures, re-probed and re-exposed as necessary. Tat56 based hairpin blots were probed with 5' CTG CTT GTA CCA ATT GCT A(B); a 19 base, 3' biotin end-labeled, DNA oligonucleotide probe (Sigma-Genosys) designed to bind the guide strand of the liberated siRNA (processed product). Each blot contained a single stranded RNA (ssRNA) marker made from three synthetic RNA oligonucleotides (Proligo) of 19 (3' GAC GAA CAU GGU UAA CGA U), 21 (3' UUU GAC GAA CAU GGU UAA CGA), and 23 (3' UUU GAC GAA CAU GGU UAA CGA UA) bases. These were designed to be approximately equivalent to the anticipated sequence of the processed products for Tat56 based hairpins to more accurately indicate product length and to enable detection simultaneously with the Tat56 products. Blots were also probed with a DNA oligo specific for the U6 small RNA to estimate loading differences 5' AAC GCT TCA CGA ATT TGC GT.

### Statistical analysis

P values were determined by analysis of variance (ANOVA, Bonferroni's multiple test comparison) using Prism 4.0a.

## Competing interests

This work was done jointly by students enrolled in The school of Biotechnology and Biomedical Science at the University of New South Wales, Sydney, Australia, and employees of Johnson and Johnson Research, for Johnson and Johnson Research.

## Authors' contributions

GJM and GCF conceived the experiments. GJM and YY performed the experiments. GJM, ML and GCF analyzed and interpreted the results. GJM and ML wrote the manuscript. All authors have read and approved the final manuscript.

## Supplementary Material

Additional file 1 **Survey and sequence details**. This file contains a list of the studies surveyed (for stem length and loop sequence), and detailed sequence information for all shRNAs used in this study. Click here for file

## References

[B1] Zeng Y Cullen BR Efficient processing of primary microRNA hairpins by Drosha requires flanking non-structured RNA sequences J Biol Chem 2005 280 30 27595 27603 10.1074/jbc.M504714200 15932881

[B2] Kim VN MicroRNA precursors in motion: exportin-5 mediates their nuclear export Trends Cell Biol 2004 14 4 156 159 10.1016/j.tcb.2004.02.006 15134074

[B3] Zeng Y Cullen BR Structural requirements for pre-microRNA binding and nuclear export by Exportin 5 Nucleic Acids Res 2004 32 16 4776 4785 10.1093/nar/gkh824 15356295 PMC519115

[B4] Schwarz DS Hutvagner G Du T Xu Z Aronin N Zamore PD Asymmetry in the assembly of the RNAi enzyme complex Cell 2003 115 2 199 208 10.1016/S0092-8674(03)00759-1 14567917

[B5] Elbashir SM Lendeckel W Tuschl T RNA interference is mediated by 21-and 22-nucleotide RNAs Genes Dev 2001 15 2 188 200 10.1101/gad.862301 11157775 PMC312613

[B6] Provost P Dishart D Doucet J Frendewey D Samuelsson B Radmark O Ribonuclease activity and RNA binding of recombinant human Dicer EMBO J 2002 21 21 5864 5874 10.1093/emboj/cdf578 12411504 PMC131075

[B7] Brummelkamp TR Bernards R Agami R A system for stable expression of short interfering RNAs in mammalian cells Science 2002 296 5567 550 553 10.1126/science.1068999 11910072

[B8] Lee NS Dohjima T Bauer G Li H Li MJ Ehsani A Salvaterra PM Rossi JJ Expression of small interfering RNAs targeted against HIV-1 rev transcripts in human cells Nat Biotechnol 2002 20 5 500 505 11981565 10.1038/nbt0502-500

[B9] McManus M Petersen CP Haines BB Chen J Sharp PA Gene silencing using micro-RNA designed hairpins Rna 2002 8 6 842 850 10.1017/S1355838202024032 12088155 PMC1370301

[B10] Miyagishi M Taira K U6 promoter-driven siRNAs with four uridine 3' overhangs efficiently suppress targeted gene expression in mammalian cells Nat Biotechnol 2002 20 5 497 500 10.1038/nbt0502-497 11981564

[B11] Paddison PJ Caudy AA Hannon GJ Stable suppression of gene expression by RNAi in mammalian cells Proc Natl Acad Sci USA 2002 99 3 1443 1448 10.1073/pnas.032652399 11818553 PMC122210

[B12] Paul CP Good PD Winer I Engelke DR Effective expression of small interfering RNA in human cells Nat Biotechnol 2002 20 5 505 508 10.1038/nbt0502-505 11981566

[B13] Sui G Soohoo C Affar el B Gay F Shi Y Forrester WC A DNA vector-based RNAi technology to suppress gene expression in mammalian cells Proc Natl Acad Sci USA 2002 99 8 5515 5520 10.1073/pnas.082117599 11960009 PMC122801

[B14] Yu JY DeRuiter SL Turner DL RNA interference by expression of short-interfering RNAs and hairpin RNAs in mammalian cells Proc Natl Acad Sci USA 2002 99 9 6047 6052 10.1073/pnas.092143499 11972060 PMC122899

[B15] Reynolds A Leake D Boese Q Scaringe S Marshall WS Khvorova A Rational siRNA design for RNA interference Nat Biotechnol 2004 22 3 326 330 10.1038/nbt936 14758366

[B16] Yu JY Taylor J DeRuiter SL Vojtek AB Turner DL Simultaneous inhibition of GSK3alpha and GSK3beta using hairpin siRNA expression vectors Mol Ther 2003 7 2 228 236 10.1016/S1525-0016(02)00037-0 12597911

[B17] Paddison PJ Caudy AA Bernstein E Hannon GJ Conklin DS Short hairpin RNAs (shRNAs) induce sequence-specific silencing in mammalian cells Genes Dev 2002 16 8 948 958 10.1101/gad.981002 11959843 PMC152352

[B18] Miyagishi M Sumimoto H Miyoshi H Kawakami Y Taira K Optimization of an siRNA-expression system with an improved hairpin and its significant suppressive effects in mammalian cells J Gene Med 2004 6 7 715 723 10.1002/jgm.556 15241778

[B19] Jeanson-Leh L Blondeau J Galy A Optimization of short hairpin RNA for lentiviral-mediated RNAi against WAS Biochemical and Biophysical Research Communications 2007 362 2 498 503 10.1016/j.bbrc.2007.08.013 17719003

[B20] Rose SD Kim D Amarzguioui M Heidel JD Collingwood MA Davis ME Rossi JJ Behlke MA Functional polarity is introduced by Dicer processing of short substrate RNAs Nucleic Acids Res 2005 33 13 4140 4156 10.1093/nar/gki732 16049023 PMC1180746

[B21] Kim DH Behlke MA Rose SD Chang MS Choi S Rossi JJ Synthetic dsRNA Dicer substrates enhance RNAi potency and efficacy Nat Biotechnol 2005 23 2 222 226 10.1038/nbt1051 15619617

[B22] Siolas D Lerner C Burchard J Ge W Linsley PS Paddison PJ Hannon GJ Cleary MA Synthetic shRNAs as potent RNAi triggers Nat Biotechnol 2005 23 2 227 231 10.1038/nbt1052 15619616

[B23] Cullen BR Derivation and function of small interfering RNAs and microRNAs Virus Res 2004 102 1 3 9 10.1016/j.virusres.2004.01.009 15068874

[B24] Castanotto D Scherer L Targeting Cellular Genes with PCR Cassettes Expressing Short Interfering RNAs Methods Enzymol 2005 392 173 185 15644181 10.1016/S0076-6879(04)92010-1

[B25] Li L Lin X Khvorova A Fesik S Shen Y Defining the optimal parameters for hairpin-based knockdown constructs RNA 2007 13 10 1765 1774 10.1261/rna.599107 17698642 PMC1986814

[B26] Zhang H Kolb FA Brondani V Billy E Filipowicz W Human Dicer preferentially cleaves dsRNAs at their termini without a requirement for ATP EMBO J 2002 21 21 5875 5885 10.1093/emboj/cdf582 12411505 PMC131079

[B27] Vermeulen A Behlen L Reynolds A Wolfson A Marshall WS Karpilow J Khvorova A The contributions of dsRNA structure to Dicer specificity and efficiency Rna 2005 11 5 674 682 10.1261/rna.7272305 15811921 PMC1370754

[B28] Zhang H Kolb FA Jaskiewicz L Westhof E Filipowicz W Single processing center models for human Dicer and bacterial RNase III Cell 2004 118 1 57 68 10.1016/j.cell.2004.06.017 15242644

[B29] Lee MT Coburn GA McClure MO Cullen BR Inhibition of human immunodeficiency virus type 1 replication in primary macrophages by using Tat- or CCR5-specific small interfering RNAs expressed from a lentivirus vector J Virol 2003 77 22 11964 11972 10.1128/JVI.77.22.11964-11972.2003 14581533 PMC254276

[B30] Paddison PJ Silva JM Conklin DS Schlabach M Li M Aruleba S Balija V O'Shaughnessy A Gnoj L Scobie K A resource for large-scale RNA-interference-based screens in mammals Nature 2004 428 427 431 10.1038/nature02370 15042091

[B31] Zuker M Mfold web server for nucleic acid folding and hybridization prediction Nucleic Acids Res 2003 31 13 3406 3415 10.1093/nar/gkg595 12824337 PMC169194

[B32] Dykxhoorn DM Novina CD Sharp PA Killing the messenger: short RNAs that silence gene expression Nat Rev Mol Cell Biol 2003 4 6 457 467 12778125 10.1038/nrm1129

[B33] McIntyre GJ Fanning GC Design and cloning strategies for constructing shRNA expression vectors BMC Biotechnol 2006 6 1 10.1186/1472-6750-6-1 16396676 PMC1343552

[B34] Williams BR Dicing with siRNA Nat Biotechnol 2005 23 2 181 182 10.1038/nbt0205-181 15696144

[B35] Lau NC Lim LP Weinstein EG Bartel DP An abundant class of tiny RNAs with probable regulatory roles in Caenorhabditis elegans Science 2001 294 5543 858 862 10.1126/science.1065062 11679671

[B36] Chu C-Y Rana TM Potent RNAi by short RNA triggers RNA 2008 14 9 1714 1719 10.1261/rna.1161908 18658119 PMC2525957

[B37] Lee Y Jeon K Lee JT Kim S Kim VN MicroRNA maturation: stepwise processing and subcellular localization EMBO J 2002 21 17 4663 4670 10.1093/emboj/cdf476 12198168 PMC126204

[B38] Paddison PJ Cleary M Silva JM Chang K Sheth N Sachidanandam R Hannon GJ Cloning of short hairpin RNAs for gene knockdown in mammalian cells Nat Methods 2004 1 2 163 167 10.1038/nmeth1104-163 16144086

[B39] Lee Y Ahn C Han J Choi H Kim J Yim J Lee J Provost P Radmark O Kim S Kim VN The nuclear RNase III Drosha initiates microRNA processing Nature 2003 425 415 419 10.1038/nature01957 14508493

[B40] Bridge AJ Pebernard S Ducraux A Nicoulaz AL Iggo RD Induction of an interferon response by RNAi vectors in mammalian cells Nat Genet 2003 34 263 264 10.1038/ng1173 12796781

[B41] Fish RJ Kruithof EK Short-term cytotoxic effects and long-term instability of RNAi delivered using lentiviral vectors BMC Mol Biol 2004 5 9 10.1186/1471-2199-5-9 15291968 PMC514603

[B42] Hannon GJ Rossi JJ Unlocking the potential of the human genome with RNA interference Nature 2004 431 371 378 10.1038/nature02870 15372045

[B43] Bartel DP MicroRNAs: genomics, biogenesis, mechanism, and function Cell 2004 116 281 297 10.1016/S0092-8674(04)00045-5 14744438

[B44] Zeng Y Wagner EJ Cullen BR Both natural and designed micro RNAs can inhibit the expression of cognate mRNAs when expressed in human cells Mol Cell 2002 9 1327 1333 10.1016/S1097-2765(02)00541-5 12086629

[B45] Boden D Pusch O Silbermann R Lee F Tucker L Ramratnam B Enhanced gene silencing of HIV-1 specific siRNA using microRNA designed hairpins Nucleic Acids Res 2004 32 1154 1158 10.1093/nar/gkh278 14966264 PMC373410

[B46] Dickins RA Hemann MT Zilfou JT Simpson DR Ibarra I Hannon GJ Lowe SW Probing tumor phenotypes using stable and regulated synthetic microRNA precursors Nat Genet 2005 37 11 1289 1295 16200064 10.1038/ng1651

[B47] Silva JM Li MZ Chang K Ge W Golding MC Rickles RJ Siolas D Hu G Paddison PJ Schlabach MR Second-generation shRNA libraries covering the mouse and human genomes Nat Genet 2005 37 11 1281 1288 16200065 10.1038/ng1650

[B48] Zeng Y Cai X Cullen BR Use of RNA Polymerase II to Transcribe Artificial MicroRNAs Methods Enzymol 2005 392 371 380 15644193 10.1016/S0076-6879(04)92022-8

[B49] Boudreau RL Monteys AM Davidson BL Minimizing variables among hairpin-based RNAi vectors reveals the potency of shRNAs RNA 2008 14 9 1834 1844 10.1261/rna.1062908 18697922 PMC2525944

[B50] Kim VN MicroRNA biogenesis: coordinated cropping and dicing Nat Rev Mol Cell Biol 2005 6 5 376 385 15852042 10.1038/nrm1644

[B51] Lee NS Rossi JJ Control of HIV-1 replication by RNA interference Virus Res 2004 102 53 58 10.1016/j.virusres.2004.01.015 15068880

[B52] Yi R Doehle BP Qin Y Macara IG Cullen BR Overexpression of exportin 5 enhances RNA interference mediated by short hairpin RNAs and microRNAs Rna 2005 11 220 226 10.1261/rna.7233305 15613540 PMC1370710

[B53] Pusch O Boden D Silbermann R Lee F Tucker L Ramratnam B Nucleotide sequence homology requirements of HIV-1-specific short hairpin RNA Nucleic Acids Res 2003 31 6444 6449 10.1093/nar/gkg876 14602902 PMC275570

[B54] Holen T Amarzguioui M Wiiger MT Babaie E Prydz H Positional effects of short interfering RNAs targeting the human coagulation trigger Tissue Factor Nucleic Acids Res 2002 30 1757 1766 10.1093/nar/30.8.1757 11937629 PMC113209

[B55] Vlassov AV Korba B Farrar K Mukerjee S Seyhan AA Ilves H Kaspar RL Leake D Kazakov SA Johnston BH shRNAs Targeting Hepatitis C: Effects of Sequence and Structural Features, and Comparision with siRNA Oligonucleotides 2007 17 2 223 236 10.1089/oli.2006.0069 17638526

[B56] Ding H Liao G Wang H Zhou Y Asymmetrically designed siRNAs and shRNAs enhance the strand specificity and efficacy in RNAi J RNAi Gene Silencing 2008 4 1 269 280 PMC2737237 19771234

[B57] Boden D Pusch O Lee F Tucker L Shank PR Ramratnam B Promoter choice affects the potency of HIV-1 specific RNA interference Nucleic Acids Res 2003 31 5033 5038 10.1093/nar/gkg704 12930953 PMC212804

[B58] Coburn GA Cullen BR Potent and specific inhibition of human immunodeficiency virus type 1 replication by RNA interference Journal of Virology 2002 76 18 9225 9231 10.1128/JVI.76.18.9225-9231.2002 12186906 PMC136455

