# Supplementary material for: The effects of stem length and core placement on shRNA activity
Source: BMC Mol Biol. 2011 Aug 8;12:34. doi: 10.1186/1471-2199-12-34 (PMC3175162; doi:10.1186/1471-2199-12-34)
Supplement: Additional file 1 — Survey and sequence details. This file contains a list of the studies surveyed (for stem length and loop sequence), and detailed sequence information for all shRNAs used in this study. [file 1471-2199-12-34-S1.PDF]

Additional Table 1

A survey of hairpin stem lengths and loop sequences

We surveyed one hundred and one studies employing expressed shRNA (for mammalian applications) and scored the stem length and loop sequence for each hairpin. In some cases the actual stem length may differ from the designed (**Intended**) length due to a collapsed loop sequence, which results in a smaller loop and extended stem (**Alt**.).

Reference	Intended	Alt.	Loop
Arrighi *et al.*, *J Virol* **78**, 10848 (Oct, 2004).	21	**23**	9 (UUCAAGAGA)^*
Babcock *et al.*, *Mol Ther* **11**, 899 (Jun, 2005).	19	**21**	9 (UUCAAGAGA)^
Bernard *et al.*, *Oncogene* (May 9, 2005).	19	**21**	9 (UUCAAGAGA)^*
Berns *et al.*, *Nature* **428**, 431 (Mar 25, 2004).	19	-	not available**
Boden *et al.*, *Mol Ther* **9**, 396 (Mar, 2004).	21	same	6
Boden *et al.*, *Nucleic Acids Res* **32**, 1154 (2004).	21, other[#]	-	other[#]
Bot *et al.*, *Blood* (May 10, 2005).	19	**21**	9 (UUCAAGAGA)^
Caldas *et al.*, *J Med Genet* (May 20, 2005).	19*	**21**	9 (UUCAAGAGA)^*
Cao *et al.*, *J Appl Genet* **46**, 217 (2005).	21	**22**	6 (AAGCUU)^
Chan *et al.*, *Genes Dev* **19**, 196 (Jan 15, 2005).	19*	**21**	9 (UUCAAGAGA)^*
Chang *et al.*, *Gene Ther* (Mar 3, 2005).	19	**21**	9 (UUCAAGAGA)^
Chen *et al.*, *World J Gastroenterol* **11**, 831 (2005).	19	**21**	9 (UUCAAGAGA)^
Corydon *et al.*, *Mol Genet Metab* (May 28, 2005).	19	**21**	9 (UUCAAGAGA)^
Das *et al.*, *J Virol* **78**, 2601 (Mar, 2004).	19	**21**	9 (UUCAAGAGA)^
Denti *et al.*, *Mol Ther* **10**, 191 (Jul, 2004).	24, other[#]	same	5 (UGUGU)
Einav *et al.*, *FEBS Lett* **579**, 199 (Jan 3, 2005).	19	**21**	9 (UUCAAGAGA)^
Fish, Kruithof, *BMC Mol Biol* **5**, 9 (Aug 3, 2004).	19, 21, 23, 25	same	8 (CAAGCUUC)^
Fung, Demple, *Mol Cell* **17**, 463 (Feb 4, 2005).	21	**23**	9 (UUCAAGAGA)^*
Gewin *et al.*, *Genes Dev* **18**, 2269 (Sep 15, 2004).	29	-	not available**
Gonzalez *et al.*, *Mol Ther* **11**, 811 (May, 2005).	21	**23**	9 (UUUGUGUAG)
Guo *et al.*, *World J Gastroenterol* **11**, 2912 (2005).	19	**20**	6 (CUCGAG)^
Hacker *et al.*, *Gene* **341**, 227 (Oct 27, 2004).	21	**23**	9 (UUCAAGAGA)^
Hernandez-Munoz *et al.*, *PNAS.* **102**, 7635 (2005).	21	**23**	9 (UUCAAGAGA)^*
Ho *et al.*, *Exp Cell Res* **304**, 1 (Mar 10, 2005).	19	**21**	9 (UUCAAGAGA)^
Hosono *et al.*, *Gene* **348**, 157 (Mar 28, 2005).	19	**21**	9 (UUCAAGAGA)^
Hua *et al.*, *Gynecol Oncol* (May 24, 2005).	19	**21**	9 (UUCAAGAGA)^
Huang *et al.*, *FEBS Lett* **558**, 69 (Jan 30, 2004).	25	same	4
Huang, Kochanek, *Hum Gene Ther* **16**, 618 (2005).	19	**21**	9 (UUCAAGAGA)^

**Reference**	**Intended**	**Alt.**	**Loop**
Hung, Kumar, *FEBS Lett* **560**, 210 (Feb 27, 2004).	19	**21**	9 (UUCAAGAGA)^
Hurtado *et al.*, *J Mol Cell Cardiol* **38**, 647 (2005).	29	same	8 (GAAGCUUG)
Izeradjene *et al.*, *Oncogene* **24**, 2050 (2005).	28	same	8
Jacobs, de Lange, *Curr Biol* **14**, 2302 (2004).	19*	**21**	9 (UUCAAGAGA)^*
Jenke *et al.*, *Hum Gene Ther* **16**, 533 (Apr, 2005).	28, 29	same	8 (GAAGCUUG)
Kamradt *et al.*, *J Biol Chem* **280**, 11059 (2005).	19	**21**	9 (UUCAAGAGA)^
Kanda *et al.*, *Biochem Biophys Res Com* **330**, 1217 (2005)	19	**21**	9 (UUCAAGAGA)^
Kariko *et al.*, *J Immunol* **172**, 6545 (Jun 1, 2004).	21	same	4 (UUAA)
Koper-Emde *et al.*, *Biol Chem* **385**, 791 (2004).	19	same	3 (ACA)
Kudo, Sutou, *J Reprod Dev* (Apr 5, 2005).	21	**23**	9 (UUCAAGAGA)^
Kuninger *et al.*, *Hum Gene Ther* **15**, 1287 (2004).	19	**21**	9 (UUCAAGAGA)^
Laatsch *et al.*, *Eur J Cell Biol* **83**, 113 (Apr, 2004).	19	**21**	9 (UUCAAGAGA)^*
Lakka *et al.*, *Oncogene* **23**, 4681 (Jun 10, 2004).	21	same	9
Lambeth *et al.*, *BMC Biotechnol* **5**, 13 (2005).	19	**21**	9 (UUCAAGAGA)^
Langlois *et al.*, *J Biol Chem* **280**, 16949 (2005).	21	same	4 (UGCG)
Lee *et al.*, *Blood* (Apr 14, 2005).	21	same	9
Lefrancois *et al.*, *Biol Proced Online* **7**, 17 (2005).	21	-	not available**
Li *et al.*, *J Lipid Res* **46**, 220 (Feb, 2005).	21	same	9 (UUUGUGUAG)
Li *et al.*, *Biochem Biophys Res Com.* **315**, 212 (2004).	19	same	4 (UUCG)
Li *et al.*, *Oligonucleotides* **13**, 401 (2003).	21	**23**	9 (UUUGUGUAG)
Li *et al.*, *FEBS Lett* (May 21, 2005).	19	**21**	9 (UUCAAGAGA)^
Li *et al.*, *Molecular Therapy.* article in press (2005).	21	**23**	9 (UUCAAGAGA)^
Liu *et al.*, *Biochem Biophys Res Com.* **324**, 1173 (2004).	19	**21**	9 (UUCAAGAGA)^
Lu *et al.*, *Biochem Biophys Res Com.* **325**, 494 (2004).	19	**21**	9 (UUCAAGAGA)^
Lu *et al.*, *Virology* **324**, 84 (Jun 20, 2004).	19	**21**	9 (UUCAAGAGA)^
Matthess *et al.*, *Oncogene* **24**, 2973 (2005).	19	same	9 (UUCAUAUGG)
Mejillano *et al.*, *Cell* **118**, 363 (Aug 6, 2004).	19	**21**	9 (UUCAAGAGA)^*
Miller *et al.*, *Nucleic Acids Res* **32**, 661 (2004).	20, 21, 22	same	8 (GAAGCUUG)
Miyagawa *et al.*, *J Biochem (Tokyo)* **137**, 503 (2005).	21	**23**	9 (UUCAAGAGA)^
Miyagishi *et al.*, *J Gene Med* **6**, 715 (Jul, 2004).	19	**20**	11 (GUGUGCUGUCC)^
Modem *et al.*, *Nucleic Acids Res* **33**, 873 (2005).	19	same	8 (GAGUACUG)
Moore *et al.*, *J Gene Med* (Mar 9, 2005).	19	**21**	9 (UUCAAGAGA)^*
Nahreini *et al.*, *Cell Mol Neurobiol* **24**, 781 (2004).	22, 27	-	other#
Nicholson *et al.*, *Mol Ther* **11**, 638 (Apr, 2005).	22	same	8 (CAUCGAUA)
Paddison *et al.*, *Nature* **428**, 427 (Mar 25, 2004).	29	same	4
Pebernard, Iggo, *Differentiation* **72**, 103 (Mar, 2004).	19	**21**	9 (UUCAAGAGA)^
Piva *et al.*, *Blood* **105**, 1750 (Feb 15, 2005).	21	**23**	9 (UUCAAGAGA)^
Poltoratsky *et al.*, *DNA Repair (Amst)* (Jun 8, 2005).	19	**21**	9 (UUCAAGAGA)^
Pruss *et al.*, *Eur J Neurosci* **20**, 3184 (Dec, 2004).	19, 22	**21, 24**	9 (UUCAAGAGA)^
Ren *et al.*, *J Viral Hepat* **12**, 236 (May, 2005).	19, 21, 23, 24, 25	same	9 (UUCAAGAGA)^
Rotolo *et al.*, *Int J Cancer* **115**, 164 (May 20, 2005).	19	**21**	9 (UUCAAGAGA)^

**Reference**	**Intended**	**Alt.**	**Loop**
Scherer *et al.*, *Mol Ther* **10**, 597 (Sep, 2004).	21	same	9
Scherr *et al.*, *Gene Ther* **12**, 12 (Jan, 2005).	19	**21**	9 (UUCAAGAGA)^
Shah *et al.*, *Biochem Biophys Res Com* **331**, 167 (2005).	21	**22**	6 (AAGCUU)^
Song *et al.*, *Biochem Biophys Res Com* **323**, 573 (2004).	23	same	6 (UUUUGC)
Spankuch *et al.*, *J Natl Cancer Inst* **96**, 862 (2004).	21	same	6
Takabatake *et al.*, *Gene Ther* **12**, 965 (Jun, 2005).	19	**21**	9 (UUCAAGAGA)^
Takigawa *et al.*, *Microbiol Immunol* **48**, 591 (2004).	19	**21**	9 (UUCAAGAGA)^
Taniai *et al.*, *Cancer Res* **64**, 3517 (May 15, 2004).	19	**21**	9 (UUCAAGAGA)^
Tao *et al.*, *Chin Med J (Engl)* **118**, 714 (2005).	20	same	4
Tatsuka *et al.*, *Oncogene* **24**, 1122 (Feb 3, 2005).	19	**21**	9 (UUCAAGAGA)^
Tiscornia *et al.*, *PNAS* **101**, 7347 (2004).	19	**21**	9 (UUCAAGAGA)^
Unwalla *et al.*, *Nat Biotechnol* (Nov 28, 2004).	28	**30**	9 (UUCAAGAGA)^
Wakiyama *et al*, *Bioc Biophys Res Com* **331**, 1163 (2005)	21	-	other[#]
Wang *et al.*, *Int J Cancer* **112**, 994 (Dec 20, 2004).	19	**21**	9 (UACAAGAGA)
Weiss-Haljiti *et al.*, *J Biol Chem* **279**, 43273 (2004).	22	same	8 (CAAGCUUC)
Westerhout *et al.*, *Nucleic Acids Res* **33**, 796 (2005).	19	-	not available**
Wu *et al.*, *Biochem Biophys Res Com* **330**, 53 (2005).	19, 26	**21, 28**	9 (UUCAAGAGA)^, other[#]
Xu, Shrager, *J Neurosci Res* **79**, 428 (2005).	19	**21**	9 (UUCAAGAGA)^
Xu *et al.*, *J Biol Chem* **279**, 41319 (Oct 1, 2004).	26	same	10 (GAAGCUUGAG)
Yang *et al.*, *World J Gastroenterol* **11**, 498 (2005).	29	same	4, 8
Yang *et al.*, *Mol Cell Biol* **25**, 4062 (May, 2005).	19	**21**	9 (UUCAAGAGA)^
Yang, Hecht, *FEBS Lett* **576**, 221 (Oct 8, 2004).	22	**23**	6 (CUCGAG)^
Yang *et al.*, *Nucleic Acids Res* **33**, e57 (2005).	19	same	4 (UUCG)
Yogosawa *et al.*, *J Biol Chem* AOL (2005).	19	**20**	12 (GTGTGCTGTCC) ^
Yoon *et al.*, *Mol Cells* **18**, 127 (Aug 31, 2004).	19, 23	same	9*
Yoon *et al.*, *Oncogene* (Apr 4, 2005).	19	**21**	9 (UUCAAGAGA)^
Yuan *et al.*, *J Orthop Res* (May 28, 2005).	19	**21**	9 (UUCAAGAGA)^
Yuan *et al.*, *Oncogene* **24**, 3657 (May 19, 2005).	19	**21**	9 (UUCAAGAGA)^
Zeng, Cullen, *Nucleic Acids Res* **32**, 4776 (2004).	19	**21**	9 (UUCAAGAGA)^
Zhang *et al.*, *Gene Ther* (May 5, 2005).	21,23	same	6
Zhou *et al.*, *Am J Pathol* **165**, 2033 (Dec, 2004).	19	-	not available**
Zhou *et al.*, *Nucleic Acids Res* **33**, e62 (2005).	22, 23	**24, 25**	9 (UUCAAGAGA)^, other[#]

** Sequences or lengths were not specifically detailed but were implied by reference to previous publications.*

*** Sequence or length details were not available.*

# Non-specific lengths or sequences were evaluated, typically microRNA-derived stem or loop configurations.

^ Loop sequences that are at least partially self-complementary at their base yet were not acknowledged as such.

Additional Table 2

Sequences and features of the hairpins used

The name, transcript size (nt), and the sequence of the expected transcript (RNA, 5' to 3') are given for each vector. Hairpin names were generated using a general nomenclature according to the sequence they target (e.g. Tat), the position in the target of the first target / shRNA (guide strand) complementary pair (e.g. nucleotide 56 as measured from the ATG start site), the length of the stem or target complementary region (e.g. 29 bp), and any characteristic unique to the particular hairpin. Transcript sizes are estimates only due to the variable nature of pol III termination believed to incorporate between 1 and 6 'U' residues. Each shRNA incorporated an 8 – 9 nt loop sequence **NiCUCGAGNi** (shown in **bold**) and for some shRNA, an extra purine was included 5' to the first base of the sense stem strand to facilitate polymerase III transcriptional initiation (indicated in **bold**) or 3' to the final base of the anti-sense stem strand as required to prevent premature termination by an early run of 'T's (indicated in **bold**).

Name	Size	Sequence (5'-3')
Tat56-15	41	**G**AAACUGCUUGUACCA**ACUCGAGA**UGGUACAAGCAGUUU**GU**$_x$
Tat56-17	45	**G**AAACUGCUUGUACCAAU**ACUCGAGA**AUUGGUACAAGCAGUUU**GU**$_x$
Tat56-18	47	**G**AAACUGCUUGUACCAAUU**ACUCGAGA**AAUUGGUACAAGCAGUUU**GU**$_x$
Tat56-19	49	**G**AAACUGCUUGUACCAAUUG**ACUCGAGA**CAAUUGGUACAAGCAGUUU**GU**$_x$
Tat56-20	51	**G**AAACUGCUUGUACCAAUUGC**ACUCGAGA**GCAAUUGGUACAAGCAGUUU**GU**$_x$
Tat56-21	53	**G**AAACUGCUUGUACCAAUUGCU**ACUCGAGA**AGCAAUUGGUACAAGCAGUUU**GU**$_x$
Tat56-22	55	**G**AAACUGCUUGUACCAAUUGCUA**ACUCGAGA**UAGCAAUUGGUACAAGCAGUUU**GU**$_x$
Tat56-23	57	**G**AAACUGCUUGUACCAAUUGCUAU**ACUCGAGA**AUAGCAAUUGGUACAAGCAGUUU**GU**$_x$
Tat56-24	59	**G**AAACUGCUUGUACCAAUUGCUAUU**ACUCGAGA**AAUAGCAAUUGGUACAAGCAGUUU**GU**$_x$
Tat56-25	61	**G**AAACUGCUUGUACCAAUUGCUAUUG**ACUCGAGA**CAAUAGCAAUUGGUACAAGCAGUUU**GU**$_x$
Tat56-26	63	**G**AAACUGCUUGUACCAAUUGCUAUUGU**ACUCGAGA**ACAAUAGCAAUUGGUACAACAGUUU**GU**$_x$
Tat56-27	65	**G**AAACUGCUUGUACCAAUUGCUAUUGUA**ACUCGAGA**UACAAUAGCAAUUGGUACAAGCAGUUU**GU**$_x$
Tat56-28	67	**G**AAACUGCUUGUACCAAUUGCUAUUGUAA**ACUCGAGA**UUACAAUAGCAAUUGGUACAAGCAGUUU**GU**$_x$

Name	Size	Sequence (5'-3')
Tat56-29	69	**G**AAACUGCUUGUACCAAUUGCUAUUGUAAA**ACUCGAGA**UUUACAAUAGCAAUUGGUACAAGCAGUUU**GU**$_x$
Tat56-33 [1]	77	**G**AAACUGCUUGUACCAAUUGCUAUUGUAAAGAGU**ACUCGAGA**ACUCUUUACAAUAGCAAUUGGUACAAGCAGUUU**GU**$_x$
Tat56-37 [1, 2]	85	**G**AAACUGCUUGUACCAAUUGCUAUUGUAAAGAGUUUGC**UCUCGAGU**GCAAACUCUUUACAAUAGCAAUUGGUACAAGCAGUUU**GU**$_x$
Tat56-41 [1]	93	**G**AAACUGCUUGUACCAAUUGCUAUUGUAAAGAGUUUGCUUUC**ACUCGAGA**GAAAGCAAACUCUUUACAAUAGCAAUUGGUACAAGCAGUUU**GU**$_x$
Vpu10-19	49	**G**AUAAUAGUAGCAAUAGUAG**ACUCGAGA**CUACUAUUGCUACUAUUAU**GU**$_x$
Vpu10-21	53	**G**AUAAUAGUAGCAAUAGUAGCA**ACUCGAGA**UGCUACUAUUGCUACUAUUAU**GU**$_x$
Vpu10-23	57	**G**AUAAUAGUAGCAAUAGUAGCAUU**ACUCGAGA**AAUGCUACUAUUGCUACUAUUAU**GU**$_x$
Vpu10-25	61	**G**AUAAUAGUAGCAAUAGUAGCAUUAG**ACUCGAGA**CUAAUGCUACUAUUGCUACUAUUAU**GU**$_x$
Vpu10-27	65	**G**AUAAUAGUAGCAAUAGUAGCAUUAGUA**ACUCGAGA**UACUAAUGCUACUAUUGCUACUAUUAU**GU**$_x$
Vpu10-29	69	**G**AUAAUAGUAGCAAUAGUAGCAUUAGUAGU**ACUCGAGA**ACUACUAAUGCUACUAUUGCUACUAUUAU**GU**$_x$
Vpu51-19	49	**G**AGCAAUAGUUGUGUGGUCC**ACUCGAGA**GGACCACACAACUAUUGCU**GU**$_x$
Vpu51-21	53	**G**AGCAAUAGUUGUGUGGUCCAU**ACUCGAGA**AUGGACCACACAACUAUUGCU**GU**$_x$
Vpu51-23	57	**G**AGCAAUAGUUGUGUGGUCCAUAG**ACUCGAGA**CUAUGGACCACACAACUAUUGCU**GU**$_x$
Vpu51-25	61	**G**AGCAAUAGUUGUGUGGUCCAUAGUA**ACUCGAGA**UACUAUGGACCACACAACUAUUGCU**GU**$_x$
Vpu51-27	65	**G**AGCAAUAGUUGUGUGGUCCAUAGUAAU**ACUCGAGA**AUUACUAUGGACCACACAACUAUUGCU**GU**$_x$
Vpu51-29	69	**G**AGCAAUAGUUGUGUGGUCCAUAGUAAUCA**ACUCGAGA**UGAUUACUAUGGACCACACAACUAUUGCU**GU**$_x$
Vpu127-19	49	**G**GAUAGACUAAUAGAAAGAG**ACUCGAGA**CUCUUUCUAUUAGUCUAUC**GU**$_x$
Vpu127-21	53	**G**GAUAGACUAAUAGAAAGAGCA**ACUCGAGA**UGCUCUUUCUAUUAGUCUAUC**GU**$_x$
Vpu127-23	57	**G**GAUAGACUAAUAGAAAGAGCAGA**ACUCGAGA**UCUGCUCUUUCUAUUAGUCUAUC**GU**$_x$
Vpu127-25	61	**G**GAUAGACUAAUAGAAAGAGCAGAAG**ACUCGAGA**CUUCUGCUCUUUCUAUUAGUCUAUC**GU**$_x$
Vpu127-27	65	**G**GAUAGACUAAUAGAAAGAGCAGAAGAC**ACUCGAGA**GUCUUCUGCUCUUUCUAUUAGUCUAUC**GU**$_x$
Vpu127-29	69	**G**GAUAGACUAAUAGAAAGAGCAGAAGACAG**ACUCGAGA**CUGUCUUCUGCUCUUUCUAUUAGUCUAUC**GU**$_x$
Vpu158-17	45	**G**GCAAUGAGAGUGAAGGA**ACUCGAGA**UCCUUCACUCUCAUUGC**GU**$_x$
Vpu158-18	47	**G**GCAAUGAGAGUGAAGGAG**ACUCGAGA**CUCCUUCACUCUCAUUGC**GU**$_x$

**Name**	**Size**	**Sequence (5’-3’)**
Vpu158-19	49	**G**GCAAUGAGAGUGAAGGAGA**ACUCGAGA**UCUCCUUCACUCUCAUUGC**GU**$_x$
Vpu158-21	53	**G**GCAAUGAGAGUGAAGGAGAAG**ACUCGAGA**CUUCUCCUUCACUCUCAUUGC**GU**$_x$
Vpu158-23	57	**G**GCAAUGAGAGUGAAGGAGAAGUA**ACUCGAGA**UACUUCUCCUUCACUCUCAUUGC**GU**$_x$
Vpu158-25	61	**G**GCAAUGAGAGUGAAGGAGAAGUAUC**ACUCGAGA**GAUACUUCUCCUUCACUCUCAUUGC**GU**$_x$
Vpu158-27	65	**G**GCAAUGAGAGUGAAGGAGAAGUAUCAG**ACUCGAGA**CUGAUACUUCUCCUUCACUCUCAUUGC**GU**$_x$
Vpu158-29	69	**G**GCAAUGAGAGUGAAGGAGAAGUAUCAGCA**ACUCGAGA**UGCUGAUACUUCUCCUUCACUCUCAUUGC**GU**$_x$
Tat3-21	51	GGAGCCAGUAGAUCCUAGACU**ACUCGAGA**AGUCUAGGAUCUACUGGCUCC**U**$_x$
Tat3-29	68	GGAGCCAGUAGAUCCUAGACUAGAGCCCU**ACUCGAGA**AGGGCUCUAGUCUAGGAUCUACUGGCUCC**U**$_x$
Tat22-21	51	CUAGAGCCCUGGAAGCAUCCA**ACUCGAGA**UGGAUGCUUCCAGGGCUCUAG**U**$_x$
Tat22-29	68	CUAGAGCCCUGGAAGCAUCCAGGAAGUCA**ACUCGAGA**UGACUUCCUGGAUGCUUCCAGGGCUCUAG**U**$_x$
Tat24-21	52	AGAGCCCUGGAAGCAUCCAGG**ACUCGAGA**CCUGGAUGCUUCCAGGGCUCU**AU**$_x$
Tat24-29	69	AGAGCCCUGGAAGCAUCCAGGAAGUCAGC**ACUCGAGA**GCUGACUUCCUGGAUGCUUCCAGGGCUCU**AU**$_x$
Tat94-21	53	**G**UUUCAUUGCCAAGUUUGUUUC**GCUCGAGG**GAAACAAACUUGGCAAUGAAA**GU**$_x$
Tat94-29	69	**G**UUUCAUUGCCAAGUUUGUUUCAUAACAAA**GCUCGAGG**UUUGUUAUGAAACAAACUUGGCAAUGAAA**GU**$_x$
Tat144-21	51	GCAGGAAGAAGCGGAGACAGC**ACUCGAGA**GCUGUCUCCGCUUCUUCCUGC**U**$_x$
Tat144-29	68	GCAGGAAGAAGCGGAGACAGCGACGAAGA**ACUCGAGA**UCUUCGUCGCUGUCUCCGCUUCUUCCUGC**U**$_x$
Tat165-21	51	GACGAAGAGCUCAUCAGAACA**ACUCGAGA**UGUUCUGAUGAGCUCUUCGUC**U**$_x$
Tat165-29	67	GACGAAGAGCUCAUCAGAACAGUCAGACU**ACUCGAGA**AGUCUGACUGUUCUGAUGAGCUCUUCGUC**U**$_x$
Tat181-21	53	**G**AACAGUCAGACUCAUCAAGCU**GCUCGAGG**AGCUUGAUGAGUCUGACUGUU**GU**$_x$
Tat181-29	69	**G**AACAGUCAGACUCAUCAAGCUUCUCUAUC**GCUCGAGG**GAUAGAGAAGCUUGAUGAGUCUGACUGUU**GU**$_x$
Tat187-21	53	**G**CAGACUCAUCAAGCUUCUCUA**GCUCGAGG**UAGAGAAGCUUGAUGAGUCUG**GU**$_x$
Tat187-29	69	**G**CAGACUCAUCAAGCUUCUCUAUCAAAGCA**GCUCGAGG**UGCUUUGAUAGAGAAGCUUGAUGAGUCUG**GU**$_x$
(Tat56-)19+10	69	**G**AAACUGCUUGUACCAAUUGGAUAACAUUU**ACUCGAGA**AAAUGUUAUCCAAUUGGUACAAGCAGUUU**GU**$_x$
(Tat56-)21+8	69	**G**AAACUGCUUGUACCAAUUGCUUAACAUUU**ACUCGAGA**AAAUGUUAAGCAAUUGGUACAAGCAGUUU**GU**$_x$

Name	Size	Sequence (5'-3')
(Tat56-)23+6	69	**G**AAACUGCUUGUACCAAUUGCUAUACAUUU**ACUCGAGA**AAAUGUAUAGCAAUUGGUACAAGCAGUUU**GU**$_x$
(Tat56-)25+4	69	**G**AAACUGCUUGUACCAAUUGCUAUUGAUUU**ACUCGAGA**AAAUCAAUAGCAAUUGGUACAAGCAGUUU**GU**$_x$
(Tat56-)27+2	69	**G**AAACUGCUUGUACCAAUUGCUAUUGUAUU**ACUCGAGA**AAUACAAUAGCAAUUGGUACAAGCAGUUU**GU**$_x$
(Tat56-)2+27	69	**G**UUACUGCUUGUACCAAUUGCUAUUGUAAA**ACUCGAGA**UUUACAAUAGCAAUUGGUACAAGCAGUAA**GU**$_x$
(Tat56-)4+25	69	**G**UUUGUGCUUGUACCAAUUGCUAUUGUAAA**ACUCGAGA**UUUACAAUAGCAAUUGGUACAAGCACAAA**GU**$_x$
(Tat56-)6+23	69	**G**UUUGACCUUGUACCAAUUGCUAUUGUAAA**ACUCGAGA**UUUACAAUAGCAAUUGGUACAAGGUCAAA**GU**$_x$
(Tat56-)8+21	69	**G**UUUGACGAUGUACCAAUUGCUAUUGUAAA**ACUCGAGA**UUUACAAUAGCAAUUGGUACAUCGUCAAA**GU**$_x$
(Tat56-)10+19	69	**G**UUUGACGAACUACCAAUUGCUAUUGUAAA**ACUCGAGA**UUUACAAUAGCAAUUGGUAGUUCGUCAAA**GU**$_x$
Tat59-21	53	**G**CUGCUUGUACCAAUUGCUAUU**ACUCGAGA**AAUAGCAAUUGGUACAAGCAG**GU**$_x$
Tat59-21 (0-21-8)	69	**G**CUGCUUGUACCAAUUGCUAUUCAUUUGGC**ACUCGAGA**GCCAAAUGAAUAGCAAUUGGUACAAGCAG**GU**$_x$
Tat59-21 (1-21-7)	69	**G**UCUGCUUGUACCAAUUGCUAUUCAUUUGG**ACUCGAGA**CCAAAUGAAUAGCAAUUGGUACAAGCAGA**GU**$_x$
Tat59-21 (2-21-6)	69	**G**UUCUGCUUGUACCAAUUGCUAUUCAUUUG**ACUCGAGA**CAAAUGAAUAGCAAUUGGUACAAGCAGAA**GU**$_x$
Tat59-21 (3-21-5)	69	**G**UUUCUGCUUGUACCAAUUGCUAUUCAUUU**ACUCGAGA**AAAUGAAUAGCAAUUGGUACAAGCAGAAA**GU**$_x$
Tat59-21 (4-21-4)	69	**G**GUUUCUGCUUGUACCAAUUGCUAUUCAUU**ACUCGAGA**AAUGAAUAGCAAUUGGUACAAGCAGAAAC**GU**$_x$
Tat59-21 (5-21-3)	69	**G**AGUUUCUGCUUGUACCAAUUGCUAUUCAU**ACUCGAGA**AUGAAUAGCAAUUGGUACAAGCAGAAACU**GU**$_x$
Tat59-21 (6-21-2)	69	**G**GAGUUUCUGCUUGUACCAAUUGCUAUUCA**ACUCGAGA**UGAAUAGCAAUUGGUACAAGCAGAAACUC**GU**$_x$
Tat59-21 (7-21-1)	69	**G**GGAGUUUCUGCUUGUACCAAUUGCUAUUC**ACUCGAGA**GAAUAGCAAUUGGUACAAGCAGAAACUCC**GU**$_x$
Tat59-21 (8-21-0)	69	**G**CGGAGUUUCUGCUUGUACCAAUUGCUAUU**ACUCGAGA**AAUAGCAAUUGGUACAAGCAGAAACUCCG**GU**$_x$
Tat56-23+6 (v2)	69	**G**AAACUGCUUGUACCAAUUGCUAUGGGGGG**ACUCGAGA**CCCCCCAUAGCAAUUGGUACAAGCAGUUU**GU**$_x$
Tat56-23+6 (v3)	69	**G**AAACUGCUUGUACCAAUUGCUAUCCCCCC**ACUCGAGA**GGGGGGAUAGCAAUUGGUACAAGCAGUUU**GU**$_x$
Tat56-23+6 (v4)	69	**G**AAACUGCUUGUACCAAUUGCUAUAUAUAU**ACUCGAGA**AUAUAUAUAGCAAUUGGUACAAGCAGUUU**GU**$_x$
Tat56-23+6 (v5)	69	**G**AAACUGCUUGUACCAAUUGCUAUGCGCGC**ACUCGAGA**GCGCGCAUAGCAAUUGGUACAAGCAGUUU**GU**$_x$
Tat56-23+6 (v6)	69	**G**AAACUGCUUGUACCAAUUGCUAUGACUGU**ACUCGAGA**ACAGUCAUAGCAAUUGGUACAAGCAGUUU**GU**$_x$
Tat56-23+6 (v7)	69	**G**AAACUGCUUGUACCAAUUGCUAUACAGUC**ACUCGAGA**GACUGUAUAGCAAUUGGUACAAGCAGUUU**GU**$_x$

**Name**	**Size**	**Sequence (5'-3')**
Tat56-24 (23+1)	59	**G**AAACUGCUUGUACCAAUUGCUAUA**ACUCGAGA**UAUAGCAAUUGGUACAAGCAGUUU**GU**$_x$
Tat56-25 (23+2)	61	**G**AAACUGCUUGUACCAAUUGCUAUAC**ACUCGAGA**GUAUAGCAAUUGGUACAAGCAGUUU**GU**$_x$
Tat56-26 (23+3)	63	**G**AAACUGCUUGUACCAAUUGCUAUACA**ACUCGAGA**UGUAUAGCAAUUGGUACAAGCAGUUU**GU**$_x$
Tat56-27 (23+4)	65	**G**AAACUGCUUGUACCAAUUGCUAUACAU**ACUCGAGA**AUGUAUAGCAAUUGGUACAAGCAGUUU**GU**$_x$
Tat56-28 (23+5)	67	**G**AAACUGCUUGUACCAAUUGCUAUACAUU**ACUCGAGA**AAUGUAUAGCAAUUGGUACAAGCAGUUU**GU**$_x$
Tat56-33 (23+10) [2]	77	**G**AAACUGCUUGUACCAAUUGCUAUACAUUUGUCA**UCUCGAGU**UGACAAAUGUAUAGCAAUUGGUACAAGCAGUUU**GU**$_x$
Tat56-37 (23+14) [2]	85	**G**AAACUGCUUGUACCAAUUGCUAUACAUUUGUCAAACG**UCUCGAGU**CGUUUGACAAAUGUAUAGCAAUUGGUACAAGCAGUUU**GU**$_x$
Tat56-41 (23+18)	93	**G**AAACUGCUUGUACCAAUUGCUAUACAUUUGUCAAACGAAAG**ACUCGAGA**CUUUCGUUUGACAAAUGUAUAGCAAUUGGUACAAGCAGUUU**GU**$_x$

1 These hairpins were not 100 % matched to the target. The nucleotide at stem position 30 was altered to a 'G' (in the upper strand, with a corresponding C in the lower strand) to prevent a run of 'T's that would have resulted in premature termination of transcription.

2 Due to a cloning error these hairpins had 'U' splitter bases instead of the 'A' splitter bases.

^ These transcripts may terminate prematurely due to an early run of 4 or more 'U' bases.

Ux: It was expected that pol III termination would add a variable number of 'U's to the 3' end of each hairpin, the exact number of which was unknown as there are conflicting reports of anywhere from 1 – 6 residues being added (2 U's [15, 16, 28], ≤ 4 U's [20], 4 U's [18, 22], ≤ 5 U's [21, 36], 4 – 6 U's [56]).

Target sequences

The fluorescent reporters used in this study were assembled with following target domains derived from the common HIV-1 laboratory strain, NL4-3 [Genbank:**AF324493**]. n.b. each of the following target domains was fused immediately downstream of GFP with several stop codons placed between the domains.

Tat x12 (both exons fused) **(261 bp)**

(positions **56** and **59** indicated in bold)

atggagccagtagatcctagactagagccctggaagcatccaggaagtcagccta**A**aa**C**tgcttgta
ccaattgctattgtaaaaagtgttgctttcattgccaagtttgtttcatgacaaaagccttaggcatctcctat
ggcaggaagaagcggagacagcgacgaagagctcatcagaacagtcagactcatcaagcttctcta
tcaaagcaacccacctcccaatcccgaggggacccgacaggcccgaaggaatag

Vpu (246 bp)

(positions **10**, **51**, **127**, and **158** indicated in bold)

atgcaacct**A**taatagtagcaatagtagcattagtagtagcaataataat**A**gcaatagttgtgtggtccat
agtaatcatagaatataggaaaatattaagacaaagaaaaatagacaggttaatt**G**atagactaataga
aagagcagaagacagtg**G**caatgagagtgaaggagaagtatcagcacttgtggagatgggggtgg
aaatggggcaccatgctccttgggatattgatgatctgtag

Vif (579 bp)

(used for the AsRed-1 fused off-target control reporter)

atggaaaacagatggcaggtgatgattgtgtggcaagtagacaggatgaggattaacacatggaaaa
gattagtaaaacaccatatgtatatttcaaggaaagctaaggactggttttatagacatcactatgaaagt
actaatccaaaaataagttcagaagtacacatcccactaggggatgctaaattagtaataacaacatatt
ggggtctgcatacaggagaaagagactggcatttgggtcagggagtctccatagaatggaggaaaaa
gagatatagcacacaagtagaccctgacctagcagaccaactaattcatctgcactattttgattgtttttc
agaatctgctataagaaataccatattaggacgtatagttagtcctaggtgtgaatatcaagcaggacat
aacaaggtaggatctctacagtacttggcactagcagcattaataaaaccaaaacagataaagccacc
tttgcctagtgttaggaaactgacagaggacagatggaacaagccccagaagaccaagggccacag
agggagccatacaatgaatggacactag
